# Fostering International Collaborations to Inform Nursing and Midwifery Policy: A Caribbean Initiative

**DOI:** 10.1111/inr.70081

**Published:** 2025-08-12

**Authors:** Eileen T. Lake, Adelais Markaki, Oscar Ocho, Tina J. Kavukattu, Denise Bryant‐Lukosius, Jody R. Lori, Dawn Munroe, Susan M. Walsh, Edwin Bolastig, Bruna Moreno Dias, Vishwanath Partapsingh, Carmen Alvarez, Pauline Anderson‐Johnson, Monique Lynch, Jaime Wynter‐Hewitt

**Affiliations:** ^1^ PAHO/WHO Collaborating Centre for Nursing and Midwifery Leadership, School of Nursing University of Pennsylvania Philadelphia USA; ^2^ PAHO/WHOCC for International Nursing, School of Nursing The University of Alabama at Birmingham Birmingham USA; ^3^ PAHO/WHO Collaborating Centre in Nursing Policies and Leadership, The UWI School of Nursing, St. Augustine, Faculty of Medical Sciences The University of the West Indies Saint Augustine Trinidad and Tobago; ^4^ Global Health Leadership Office, College of Nursing University of Illinois Chicago Chicago USA; ^5^ PAHO/WHO Collaborating Centre in Primary Care Nursing and Health Human Resources, School of Nursing, Faculty of Health Sciences McMaster University Hamilton Canada; ^6^ School of Nursing University of Michigan Ann Arbor USA; ^7^ PAHO/WHO Collaborating Centre for Nursing and Midwifery Development in the Caribbean, The UWI School of Nursing, Mona, Faculty of Medical Sciences The University of the West Indies Saint Augustine Trinidad and Tobago; ^8^ PAHO/WHO Collaborating Centre for International Nursing Development in Primary Health Care, College of Nursing University of Illinois Chicago Chicago USA; ^9^ Human Resources for Health Caribbean Subregional Program Coordination, Pan American Health Organization Bridgetown Barbados; ^10^ Department of Health Systems and Services Pan American Health Organization Bridgetown Barbados; ^11^ Health Systems and Services, Office for Barbados and the Eastern Caribbean Countries, Caribbean Subregional Program Coordination Pan American Health Organization Bridgetown Barbados; ^12^ Department of Family and Community Health, School of Nursing University of Pennsylvania Philadelphia USA; ^13^ The UWI School of Nursing, Mona, Faculty of Medical Sciences The University of the West Indies Saint Augustine Trinidad and Tobago

**Keywords:** Advanced practice nursing, Caribbean region, health services, human resources for health, International Collaboration, midwifery, networks, nursing education, Pan American Health Organization, World Health Organization

## Abstract

**Aim:**

This initiative aimed to contribute to the Pan American Health Organization/World Health Organization's (PAHO/WHO) strategic goals for nursing and midwifery workforce development in the Caribbean by establishing a collaboration to streamline processes to collect comprehensive region‐wide data and reduce respondent burden.

**Background:**

The Caribbean region faces persistent challenges in nursing and midwifery workforce development due to high migration rates, workforce shortages, and limited regional data collection. Seven PAHO/WHO Collaborating Centers (CCs) for Nursing and Midwifery were tasked separately with Terms of Reference to support the human resources for health (HRH)‐related Sustainable Development Goals of Caribbean countries.

**Sources of evidence:**

PAHO‐tasked Terms of Reference for CC activities and strategic priorities in the Caribbean.

**Discussion:**

Using Donabedian's structure–process–outcome model, we described the development, implementation, and impact of this initiative. Structural components included the Pan American Collaborating Centers for Nursing and Midwifery (PANMCC) network, formal leadership, PAHO support, team commitment and expertise, and resources. The process emphasized regular communication, stakeholder engagement, and a collaborative approach to survey design and distribution. Outcomes included the successful development and implementation of a comprehensive survey, improved data quality, reduced respondent burden, and shared learning. Unexpected benefits were the strengthening of the PANMCC network and further opportunities for collaboration.

**Conclusion:**

The success of this initiative was attributed to structures and processes that set the foundation for effective communication, collaboration, and synergies among the CCs to achieve project goals.

**Implications for nursing policy and practice:**

This international collaboration showcased the importance of strong leadership, mutual commitment, and unwavering support from the PANMCC network. The findings demonstrate the potential of coordinated efforts to inform nursing and midwifery HRH policy and practice in resource‐constrained regions.

## 1 Aim

The aim of this initiative was to optimize the impact of Pan American Health Organization/World Health Organization (PAHO/WHO) Collaborating Centers (CCs) on nursing and midwifery workforce planning, development, and investment in the Caribbean by establishing a multicenter collaboration to streamline processes to collect comprehensive region‐wide data and reduce respondent burden. The ultimate goal was to contribute to PAHO/WHO strategic goals related to human resources for health (HRH) in the Caribbean.

## 2 Background

The PAHO/WHO CCs for Nursing and Midwifery contribute to the optimization of the nursing and midwifery workforce in Latin America and the Caribbean through varied policy, practice, education, and research initiatives. PAHO tasks these centers with Terms of Reference (TORs) to inform the development and implementation of HRH strategies that strengthen the nursing and midwifery workforce. The Caribbean region faces significant HRH challenges, particularly a critical shortage of nurses and midwives. This shortage is exacerbated by high rates of migration to developed countries such as Canada, the United States, and the United Kingdom (Rolle Sands et al. 2020). Subsequently, migration has left many Caribbean countries with nursing vacancy rates averaging 40%, posing a severe threat to the stability and effectiveness of health systems (Edwards et al. 2021; Rolle Sands et al. 2020). The COVID‐19 pandemic has further underscored the need for resilient health systems supported by a robust health workforce. To address these HRH challenges, PAHO (2023a) approved the “Policy on the Health Workforce 2030: Strengthening Human Resources for Health to Achieve Resilient Health Systems.” This policy aims to guide PAHO member states to develop and implement strategies to strengthen their health workforce, recover public health gains, and advance toward achieving sustainable development goals (SDGs), especially related to governance, regulatory mechanisms, interprofessional health teams, workforce capacity building and work conditions.

In line with this policy, initiatives such as the training and implementation of *Health Labour Market Analysis* and the *Human Resources for Resilient Health Systems Caribbean Roadmap* (PAHO 2024) have been pivotal in strengthening HRH strategies in the region. These efforts aim to ensure that health workforce planning, development, and investment are aligned with the SDGs related to health. In this article, we use the term health workforce development to also include planning and investment, consistent with recommendations of the International Council of Nurses (2024) and PAHO (2022). PAHO strategies recommended for the Caribbean include: (1) expanding nursing roles to meet evolving healthcare needs and (2) improving the supply of nurses and midwives by ensuring the quality and accessibility of education and training programs (PAHO 2023b).

Both of the above PAHO strategies are embedded in the TORs for Pan American Nursing and Midwifery CCs (PANMCC, n.d.). The CCs bolster these strategies by serving as extensions of the WHO to address health policy and practice worldwide. These centers, hosted by academic and research institutions globally, provide technical expertise, conduct research, and build local capacity to address pressing health challenges. Their collaborative approach ensures that health innovations and strategies are contextually relevant and scalable (WHO n.d.). By fostering collaboration among regional and international partners, the CCs aim to offer sustainable solutions that enhance the health workforce and improve health in the Caribbean.

## 3 Rationale and Organizational Participants

Developing and sustaining nursing and midwifery HRH is a global issue that requires international collaboration. The WHO's (2020) *State of the World's Nursing* report emphasizes the need to expand efforts and investments to strengthen the workforce, underlying the importance of collaboration in nursing education, research, and practice. In addition, the *Global Strategic Directions for Nursing and Midwifery 2021–2025* (WHO 2021) and the PAHO (2022) document *“The Strategic Importance of National Investment in Nursing Professionals in the Region of the Americas*” present policy priorities and recommendations, which also include collaboration in research and practice. Traditional efforts by international researchers and HRH‐relevant centers to inform regional health policy often fall short due to fragmentation and redundancy (WHO 2016). A multi‐center collaborative approach may address these shortcomings and more effectively achieve regional health policy goals.

The innovative collaboration presented here addresses health workforce‐related challenges in the Caribbean by bringing together key organizations and networks, each contributing unique expertise and resources. These include PAHO, an international public health agency serving as a regional office for the WHO and playing a pivotal role in improving health across the Americas (PAHO n.d.‐a). The Caribbean Subregional Program Coordination office, a vital arm of PAHO, is instrumental in advancing efforts tailored to the unique health priorities of Caribbean nations. This program aligns with the SDGs to strengthen HRH, enhance service delivery, and achieve equitable health outcomes in the region (PAHO n.d.‐b).

Additionally, the PANMCC network unites nursing and midwifery CCs across the Americas to confront shared challenges and disseminate effective solutions. By fostering multi‐institutional collaboration, the network contributes significantly to workforce development, policy advocacy, and nursing and midwifery research in the Americas (Naegle et al. 2023). This network's efforts underscore the importance of integrating nursing and midwifery perspectives into health systems to improve care delivery and health outcomes (PANMCC n.d).

### 3.1 Genesis of This Initiative

This collaborative initiative among 7 of the 13 CCs within PANMCC (see Table 1) was sparked during a November 2023 meeting to address shared priorities for nursing and midwifery workforce development in the Caribbean (PAHO 2023b). At this meeting, the seven CCs identified overlapping TORs and outreach to the same regional targets (e.g., educational and service institutions) as issues to address through enhanced CC collaboration. The meeting was co‐hosted by the PAHO/WHO Human Resources for Health Unit and the PAHO/WHO CC for International Nursing Development in Primary Health Care (University of Illinois Chicago). It brought together key stakeholders, including representatives from the Caribbean Community's (CARICOM) Regional Nursing Body (RNB), Caribbean institutions (e.g., colleges/universities, ministries of health, nursing councils, and professional organizations), and PAHO/WHO CCs. Attendees from 20 countries exchanged diverse perspectives, reinforcing the need for regional alignment in curricula, regulation, and interprofessional collaboration to improve nursing and midwifery across the Caribbean (PAHO, 2023c). Discussions focused on strengthening primary health care through nursing and midwifery education, practice, and collaboration. The meeting advanced policy recommendations to (1) boost investment in nursing and midwifery education, (2) improve regulatory frameworks, and (3) enhance workforce retention.

**TABLE 1:** Participating Collaborating Centers.

Collaborating Center's Name (WHO Reference No.)	Institution	Country
PAHO/WHO Collaborating Centre in Primary Care Nursing and Health Human Resources (CAN‐39)	McMaster University	Canada
PAHO/WHO Collaborating Centre for International Nursing (USA‐241)	University of Alabama at Birmingham	USA
PAHO/WHO Collaborating Centre for International Nursing Development in Primary Health Care (USA‐193)	University of Illinois Chicago	USA
PAHO/WHO Collaborating Centre for Research and Clinical Training in Health Promotion Nursing (USA‐283)	University of Michigan	USA
PAHO/WHO Collaborating Centre for Nursing and Midwifery Leadership (USA‐206)	University of Pennsylvania	USA
PAHO/WHO Collaborating Centre for Nursing and Midwifery Development in the Caribbean (JAM‐15)	University of West Indies–Mona	Jamaica
PAHO/WHO Collaborating Centre in Nursing Policies and Leadership (TRT‐1)	University of West Indies–St. Augustine	Trinidad and Tobago

**FIGURE 1:**
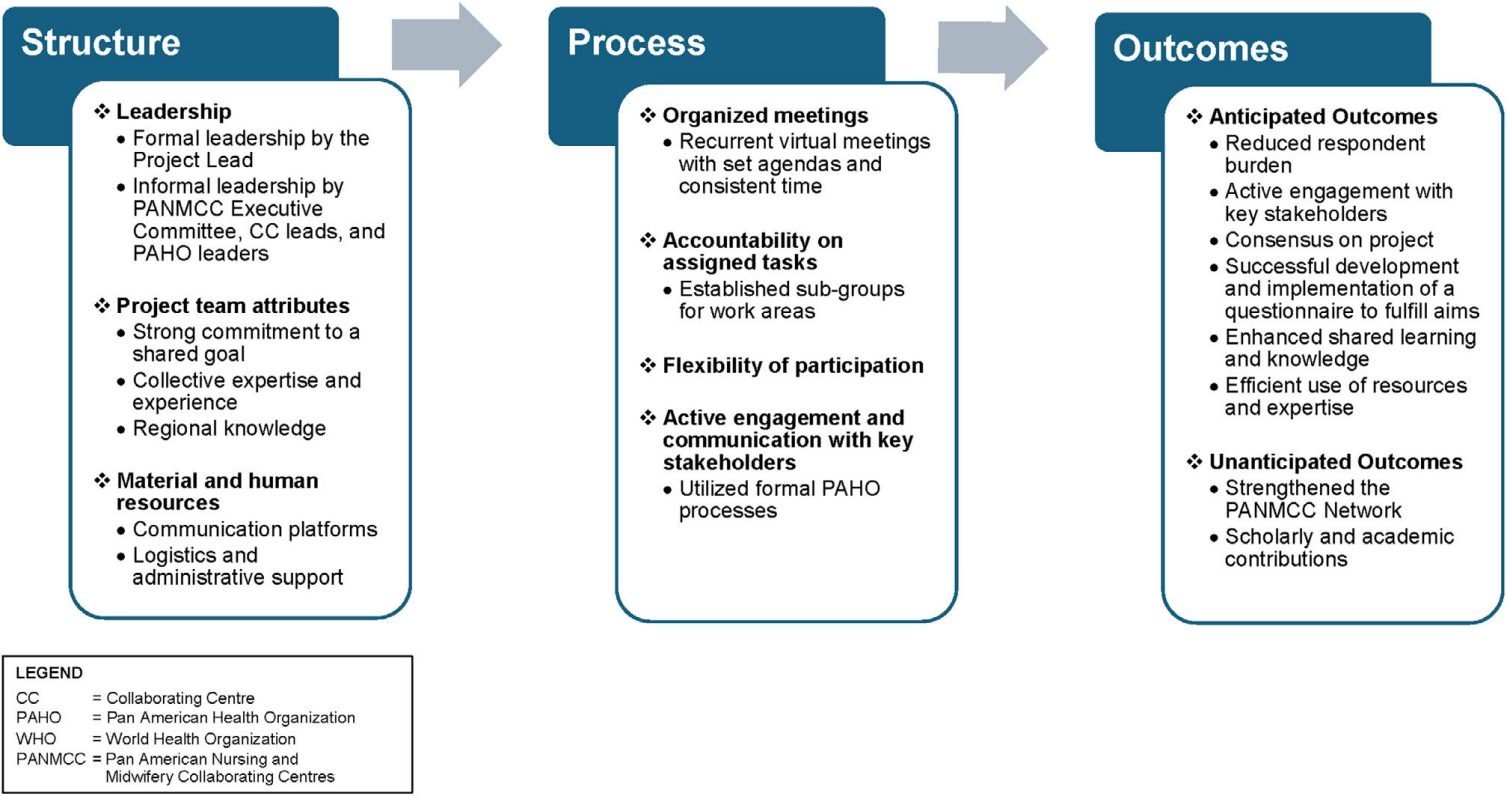
Key structure, process, and outcome elements of the Caribbean collaboration initiative.

The above event provided an impetus for targeted collaboration among seven CCs. To the best of our knowledge, no similar collaboration among CCs serving the Caribbean region has been reported, and previous efforts to collect data were fragmented and lacked the required rigor. This article describes the features of this novel, international collaboration and examines factors that contributed to its success and implementation challenges. This initiative grew organically and was not guided by a specific model. For this paper, we adopted Donabedian's (1988) structure–process–outcome model for assessing and improving healthcare quality to identify and examine the features of this collaboration. Based on this model, structures refer to physical and organizational characteristics and resources, processes refer to activities required for interactions and project completion, and outcomes refer to the project's anticipated and unanticipated effects. An overview of the key structure, process, and outcome elements of this initiative is presented in Figure 1.

### 3.2 Sources of Evidence

PAHO assigns TORs for each CC with related activities for a set designation period related to nursing role expansion, migration, health service delivery, and education/training. The TOR activities for each CC were reviewed and mapped across the PANMCC network. PAHO strategic HRH priorities in the Caribbean region were also reviewed (PAHO 2023a).

## 4 Discussion

In the following sections, we describe the structures, processes, and outcomes of our collaboration initiative. Structures relate to leadership arrangements, team characteristics, practical and human resources, and expertise required to implement the project. These structures influenced processes or the ways we collaborated and worked together to achieve project aims. Outcomes are the results of the combined effects of project structures and processes, as well as unanticipated outcomes that arose from the collaboration.

### 4.1 Collaboration Structures

**FIGURE 2:**
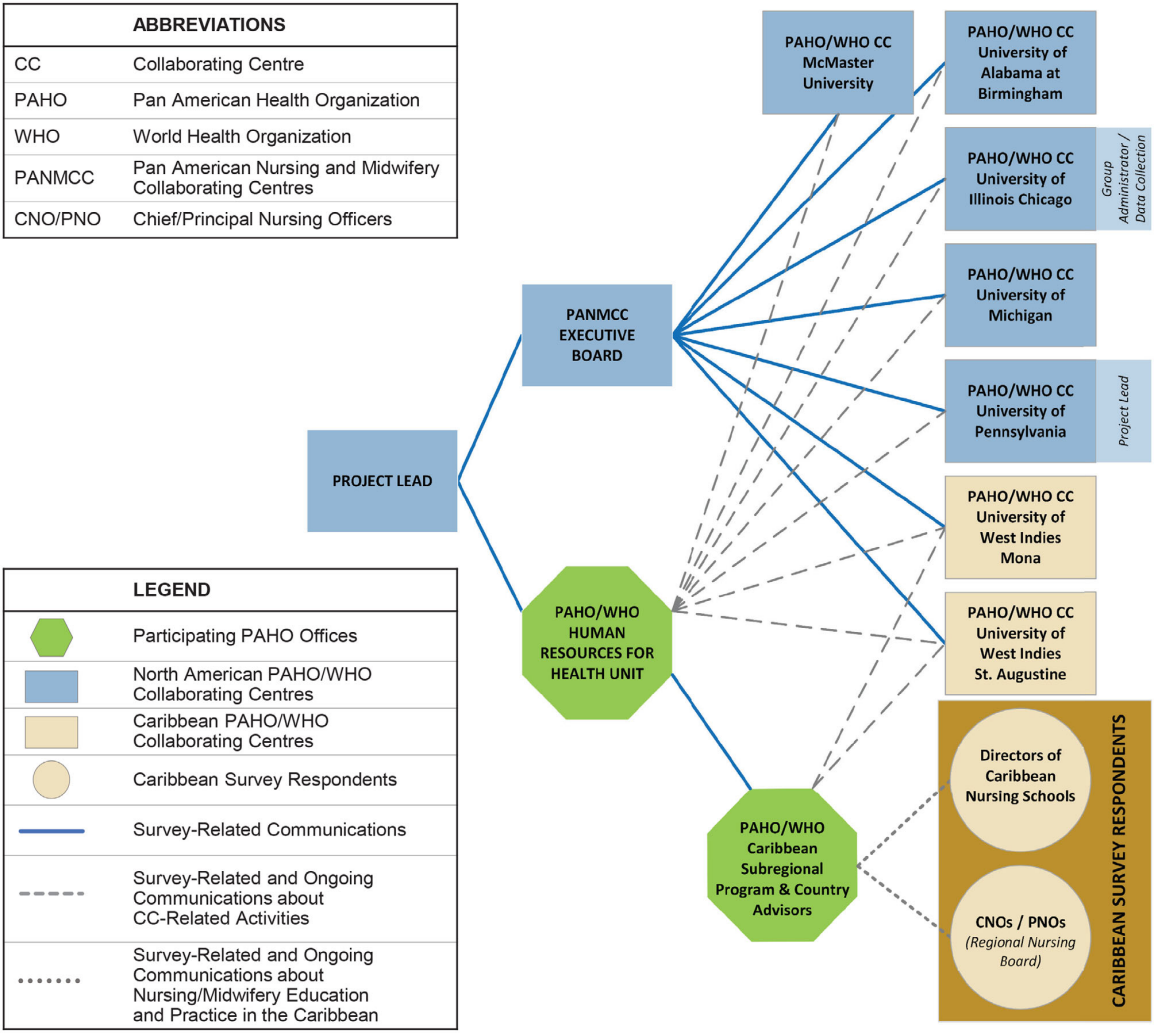
Collaboration Structure, Survey Respondent Groups, and Communication Paths.

The structural foundation of the initiative was both formal and informal leadership. The Project Lead provided leadership with the participation and support of PANMCC Executive Committee and PAHO leaders, including the Regional Advisor for Nursing and Allied Health Personnel Development, an International Consultant, the HRH Unit Chief, a Caribbean Subregional HRH Advisor, and an Eastern Caribbean Country Advisor for Health Systems. The collaboration was guided by CC leads, each bringing varied levels of resources and time commitment. A defining characteristic of the project team was a strong commitment to a shared goal and collective expertise. The project benefited from regional knowledge about nursing and midwifery education, workforce issues, and key stakeholders, particularly from Caribbean CCs, PAHO advisors, and CC leads with prior experience in the region. Additionally, the team possessed expertise in international collaborations, global health nursing, advanced practice nursing, and midwifery education. Research expertise was instrumental in developing and implementing a questionnaire as the focus of this initiative. Familiarity with PAHO policies and communication networks facilitated engagement. Effective communication platforms, including Zoom, Google Docs, and BOX, helped maintain connectivity and organize documents. A flow chart depicting the structure of the collaboration is presented in Figure 2. The group administrator role was established to prepare the questionnaire content in an online platform, maintain data files and stakeholder contact lists, monitor response rates, uphold the integrity of the survey database, and facilitate the distribution of the data.

### 4.2 Collaboration Processes

Collaboration processes included organized meetings and ongoing communication. The time commitment from the CCs was extensive, spanning November 2023 through January 2025; from survey development and distribution to review and organization of the results. The Project Lead coordinated bimonthly and ad hoc meetings, set agendas, and ensured access to relevant materials and updates via email. Team members were actively engaged by leveraging their regional knowledge and expertise to inform aspects of the project, such as recruitment, identifying key informants, and developing and piloting questionnaire items aligned with TORs. The team worked within defined timelines, ensuring accountability through consistent follow‐through on action items between meetings. The project team embraced flexibility, accommodating participation based on members’ schedules while maintaining a consistent meeting time to foster continuity.

Starting in December 2023, one‐hour‐long virtual meetings were scheduled. PAHO advisors joined the meetings beginning in February 2024 to assist with planning and the logistics of survey distribution. The meetings had a set agenda and engaged CC representatives, PAHO advisors, and other stakeholders to ensure alignment with regional priorities. Three subgroups were established based on each CC's TORs, with a charge to develop survey questions related to nursing and midwifery service, education, or advanced practice roles. Subgroups worked asynchronously to draft questions. Meeting time was used to examine survey questions for relevance and duplication of content. Time was also dedicated to developing the research protocol and institutional review board (IRB) documents for submission to the PAHO Ethics Review Committee (PAHOERC). During the meetings, lists of chief nursing or principal nursing officers (CNO/PNO) and directors of educational institutions were compiled by members using their prior contacts and lists available from the CCs located in Caribbean countries (UWI‐St. Augustine; UWI‐Mona) and PAHO.

#### 4.2.1 Survey Development

The survey was designed collaboratively to guarantee regional relevance and comprehensiveness. Each CC developed items relevant to their respective TOR activities and assigned the items to target respondents. Survey subsections included (1) technology access, (2) clinical simulation in nursing and midwifery education, (3) advanced practice nursing specialties, (4) midwifery practice, (5) evidence‐based practice, and (6) factors affecting nurse migration. The group administrator combined the items and assigned them to either an Education or a Ministry of Health/Clinical Leadership category. Subsequently, items and response options were refined for clarity and content overlap. The items were transferred to Qualtrics, a secure platform, hosted by the University of Illinois Chicago. The team reviewed and piloted the electronic version for clarity and usability prior to distribution.

#### 4.2.2 Survey Population Identification

Twenty Caribbean countries were identified with the guidance of PAHO's advisors, and CNOs/PNOs, directors of schools of nursing, and midwifery and nursing service directors from each country were included. The compiled list of the target respondents ensured comprehensive data coverage. It is important to note that within the Caribbean context, CNO/PNO refers to the highest‐ranking public sector nurse in a country. Their positions typically fall within the country's Ministry of Health, and they are responsible for oversight of nursing and midwifery practice, including regulatory policy. They may also be wholly or jointly responsible for nursing and midwifery educational policy.

#### 4.2.3 Survey Promotion and Data Collection

A PANMCC co‐chair attended the March 2024 meeting of the Caribbean's RNB to describe the project's purpose and plan, and to garner support for survey completion. Formal communication from the PAHO Subregional Program Director, prepared by the Subregional Advisor for Human Resources for Health, was circulated to PAHO/WHO Country Representatives requesting that they distribute the survey to (1) the respective CNO/PNO of each country and (2) the senior leadership of accredited nursing/midwifery schools in each country (e.g., President, Principal, Director). Data collection occurred from September to December 2024. To raise awareness of and support for the survey, the PANMCC Chair disseminated information during the seventh meeting of the Human Resources for Health Caribbean Commission. PAHO and the Caribbean CCs conducted additional outreach to increase response rates.

#### 4.2.4 Ethical Considerations

IRB approval from PAHOERC was received in April 2024 (Ref. No: PAHOERC.0733.01). The authors used generative AI (i.e., ChatGPT and Microsoft CoPilot) to prepare an initial draft of this article based on discussion points at team meetings, a colloquium presentation, the ethical protocol application, and this journal's author guidelines. The draft was revised and further developed during multiple meetings and rounds to ensure accuracy and thoroughness.

### 4.3 Collaboration Outcomes

As shown in Figure 1, the project yielded significant expected outcomes. The collaboration structure (Figure 2) enabled leadership and engagement among the CCs as well as nursing policy makers at the RNB. This structure facilitated the development and implementation of the survey because of the group's connection to relevant stakeholders. Consensus was reached on project aims, leading to the development of a comprehensive questionnaire that effectively addressed the information needs of participating CCs to complete their TORs. Decreased duplication of effort and increased collaboration between CCs resulted in the creation of a high‐quality, comprehensive dataset from the questionnaire in Qualtrics. Despite the complexity of the survey branching, the group administrator contributed to the completeness of the dataset with the creation of a streamlined, accessible online questionnaire. By monitoring survey completion and providing technical support, the administrator facilitated data collection. Team collaboration played a crucial role in cleaning and organizing the dataset for analysis.

To decrease respondent burden, the collaboration yielded a single, comprehensive survey targeting multiple stakeholders to capture workforce data in the Caribbean. The questionnaire itself was country‐relevant and context‐sensitive because of the collaboration with Caribbean colleagues. Another outcome contributing to quality data was the robust survey response rate of 100% for CNOs and 60% for educators. Finally, a relevant and comprehensive survey, with IRB approval, was developed expeditiously within six months from inception to readiness for distribution. These outcomes culminated from the synergistic efforts of the CCs and PAHO, enhanced linkages to existing networks, and resource optimization.

Beyond the expected project outcomes, the collaboration generated several unanticipated benefits. The initiative strengthened the PANMCC network by fostering deeper work relationships, enhancing teamwork, and reinforcing commitment to the network among CCs. The project's success and the positive collaborative experience have established a solid foundation for future PANMCC initiatives. Furthermore, the project created opportunities for scholarly and academic contributions, including an oral presentation at the November 2024 Pan American Nursing Research Colloquium held in Chile and the development of this article. Lastly, participation in the project enhanced shared learning and knowledge of nursing and midwifery issues, stakeholders, and networks among CC leaders in the Caribbean, equipping them with valuable insights to inform future collaborations and partnerships.

Similar to other international partnerships, there were some challenges. Member participation in meetings fluctuated due to changing workloads. Thus, time during meetings was needed to help members catch up and to ensure ongoing understanding and agreement to group decisions and follow‐up responsibilities with a clear timeline. There were also cultural and technical differences in how workforce concepts or terms were defined and in teaching and clinical skill expectations across countries. This required establishing universally accepted definitions. Differences in communication styles, compliance with existing protocols, and variable access to information technology also necessitated troubleshooting, cooperation, and coaching amongst team members.

## 5 Conclusions

Experience from this initiative contributes to recommendations for establishing future international collaborations for the nursing and midwifery workforce. Key lessons include the importance of strong leadership, mutual benefits, shared vision, sustained effort, willingness to share resources, welcoming attitude, communication, respect, and human resource capital.

The success of this initiative was attributed to several enablers which set the foundation for effective communication and collaboration among the CCs to achieve project goals: namely, leadership, motivation to collaborate, commitment, support from PAHO, flexible responsive engagement, and investment from all CCs. Capacity building was strengthened through knowledge and skills exchange and by leveraging the unique expertise of collaborators across CCs.

### 5.1 Implications for Nursing and Midwifery Policy

This initiative provides a model for future sustainable global nursing and midwifery workforce activities, achieved through partnerships, potentially maximizing the benefits for all stakeholders (Edwards et al. 2020). By identifying critical needs and fostering targeted interventions in the Caribbean, the model provides guidance for strengthening nursing and midwifery capacities. Enhanced data collection ensures that education and service improvements align with workforce realities. By sharing expertise and scarce resources through this innovative and reciprocal approach, optimum outcomes can be achieved. Lessons learned may provide a roadmap that others can use to establish effective international collaborations for nursing and midwifery workforce development.

The partnership between PAHO and CCs demonstrates the potential for international collaboration to inform policy development. As demonstrated by previous CC projects, capacity development based on reciprocal exchanges can contribute toward sustainability (Watts et al. 2020). To assess sustainability and policy impact, yearly CC activity reports should account for longitudinal project monitoring and follow‐up.

Figure 2 illustrates the complex structures and processes used to engage initiative members and stakeholders in international collaborations. Models utilizing the structure–process–outcome framework might serve to ground future international collaborations spearheaded through a network, such as PANMCC. For example, elements of this collaboration initiative aligned with the Bergen Model of Collaborative Functioning, describing the interplay of structures, processes, and outcomes to achieve the desired synergy (Corbin and Mittelmark 2008). Initiative structures were related to leadership arrangements, team characteristics, and the practical and human resources required to implement the project. These structures influenced processes or different ways the CCs collaborated to achieve project aims. As shown in Figure 1, outcomes, both expected and unanticipated, are the results of structures and processes related to the collaboration. Similarly, the Operational Model of Sustainability in Global Nursing identifies structures and processes of partnerships that lead to positive changes and sustainable outcomes (Edwards et al. 2020). These three models could be considered for future international collaborative initiatives that promote sustainable interprofessional synergies and outcomes and better facilitate coherent policy responses.

## Author Contributions

Manuscript conception: AM, CA, DBL, DM, ETL, JRL, OO, PAJ, SMW, and VAP. Initial draft: TJK. Figure: DM and TJK. Table: TJK. Abstract: AM, CA, DBL, DM, EB, ETL, JRL, JWH, ML, OO, PAJ, SMW, and TJK. Background: AM, BMD, CA, DBL, DM, EB, ETL, JRL, JWH, ML, OO, PAJ, SMW, TJK, and VAP. Discussion: AM, BMD, CA, DBL, DM, EB, ETL, JRL, JWH, ML, OO, PAJ, SMW, TJK, and VAP. Conclusions and implications: AM, CA, DBL, DM, EB, ETL, JRL, PAJ, SMW, and TJK. Editorial review of manuscript: AM, BMD, CA, DBL, DM, EB, ETL, JRL, JWH, ML, OO, PAJ, SMW, TJK, and VAP.

## Conflicts of Interest

No conflict of interest has been declared by the authors. The opinions expressed in this manuscript are the sole responsibility of the authors and do not necessarily reflect the criteria or policies of the Pan American Health Organization or of the CCs’ respective universities.
